# Frequency of basic public health services utilization by married female migrants in China: associations of social support, discrimination and sociodemographic factors

**DOI:** 10.1186/s12905-021-01482-3

**Published:** 2021-09-28

**Authors:** Xin Hu, Mei Sun, Siyuan Tang, Lisa L. Lommel

**Affiliations:** 1 No 172 Xiangya School of Nursing, Central South University, Tongzipo Road, Yuelu District, Changsha, Hunan Province 410013 People’s Republic of China; 2 Ruijin Hospital, School of Medicine, Shanghai Jiaotong University, Shanghai, People’s Republic of China; 3 Hunan Women’s Research Association, Changsha, Hunan People’s Republic of China; 4 Department of Family Health Care Nursing, School of Nursing, University of California, San Francisco, CA USA

**Keywords:** Basic public health services, Rural-to-urban, Migrant women, Social support, Discrimination

## Abstract

**Background:**

Utilization of basic public health services (BPHS) allows for disease prevention and management and is an essential component for protecting health. Disparities in utilization exist between rural-to-urban migrants and their local counterparts in China. This study sought to determine the frequency of BPHS utilization and whether social support, discrimination, and sociodemographic features were risk factors for low BPHS utilization by Chinese female migrants.

**Methods:**

Data were derived from a survey of female rural-to-urban migrants at nine work sites in Changsha, China. The association between social support, discrimination, sociodemographic factors and BPHS utilization was obtained using Chi-square and logistic regression analysis.

**Results:**

Between December 2017 and April 2018, 307 female participants completed the survey. A total of 24.7% reported having had health education, 26.1% had breast and cervical cancer screening, 27.2% had established a health care record, and 40.9% had received basic contraceptive services. Two factors were associated with the reduced likelihood of BPHS utilization: Length of migration and health record establishment (OR = 0.53; 95% CI = 0.31, 0.92) and years of education and basic contraceptive service use (OR = 0.36; 95% CI = 0.20, 0.67). The remaining six factors were associated with an increased likelihood of BPHS utilization: Living circumstances and health record establishment (OR = 2.11; 95% CI = 1.17, 3.80), health education (OR = 2.71; 95% CI = 1.51, 4.87) and cancer screening (OR = 2.38; 95% CI = 1.30, 4.36). Utilization of social support was associated with health record establishment (OR = 1.24; 95% CI = 1.06, 1.44), basic contraceptive service use (OR = 1.21; 95% CI = 1.04, 1.42) and cancer screening (OR = 1.29; 95% CI = 1.10, 1.51). Objective social support was associated with health education utilization (OR = 1.15; 95% CI = 1.04, 1.26), while subjective social support was associated with basic contraceptive service use (OR = 1.11; 95% CI = 1.05, 1.18) and cancer screening (OR = 1.10; 95% CI = 1.02, 1.17). Family location was associated with basic contraceptive service use (OR = 1.96; 95% CI = 1.12, 3.44) and migration time in Changsha was associated with basic contraceptive service use (OR = 2.24; 95% CI = 1.18, 4.27).

**Conclusions:**

Overall, there was low utilization rate for four BPHS by Chinese female migrants, and social support appears to be an important factor in this setting. Government, community, and workplace education efforts for enhancing BPHS utilization among female rural-to-urban migrants are recommended.

**Supplementary Information:**

The online version contains supplementary material available at 10.1186/s12905-021-01482-3.

## Background

Despite the growing numbers of migrants in China, the local governments in urban cities have mainly addressed only three health problems for rural-to-urban migrants: infectious diseases, reproductive health, and occupational diseases due to the effects of these problems on local residents [1]. As a result, prevention and control of non-communicable disease for rural-to-urban migrants continues to receive inadequate attention.

The National BPHS is one of very few programs that cover non-communicable disease prevention and control for rural-to-urban migrants. These services, mainly provided by community health centers (CHC), are available to all residents free of charge. BPHS are the most basic preventive health service program provided by the Chinese government to current urban and rural residents and focus on children, maternity services, the elderly, and chronic disease conditions [2]. In order to promote the development of BPHS in China, the National Health Commission issued its first detailed set of regulations and standards in October 2009. In 2019, after several revisions, the BPHS program included 31 services, with 6 of 31 services specifically for women: (1) the establishment of health records, (2) health education, (3) basic contraceptive services for married couples, (4) breast and cervical cancer screening in rural populations, (5) prenatal diagnosis evaluations, and (6) maternal health care record establishment, at least five prenatal visits, timely care within the first 12 weeks of pregnancy, and postnatal care.

Historically, rural-to-urban migrant workers have been affected by the *hukou* system [3]. In China, *hukou* defines a person’s rights for health care services, and social welfare within a specified locality [4], services are provided to citizens according to their *hukou* location and classification (rural vs. urban). For example, one study demonstrated that rural-to-urban migrants showed lower basic public health services (BPHS) utilization (30.37%) compared with local residents (43.23%) [5]. Additionally, the long-established *hukou* system is the basis for implementation of many public policies and social welfare programs [1]. A change of *hukou* status is difficult when people migrate from rural to urban settings or from one place to another [3]. Thus, social welfare and healthcare benefits in urban areas are not available to the rural-to-urban migrants, only for residents with an urban and locally registered *hukou*. However, rural migrants can now apply for a resident permit, thus allowing them to have the same rights as a local resident in an urban city; for example, they may now have the same access to BPHS and health insurance. Although rural-to-urban migrants’ basic rights while living in cities have been progressively improving by actions of the Chinese government, resident permits still cannot guarantee all rural-to-urban migrants will have the same rights as city residents. There are requirements for applying for resident permits. For example, migrants need to have lived in the city for more than six months and have a steady work contract [6]. For a cosmopolitan city like Shenzhen, the application requirements are more restricted since migrants need to have lived in the city for more than 12 months and have made 12 months of social insurance payments [6]. Therefore, there is still a gap between migrant workers' access to the same rights as urban residents in China.

According to the annual survey of rural migrant workers, there were 288.36 million rural laborers working in China in 2018 (an increase of 1.84 million compared to 2017), and 79.7% of rural migrants are married [7]. Among all rural migrants, female migrant workers accounted for 34.8%, which is a slight increase compared with 2019 (34.4%) [7]. As a result of the large and increasing population of female migrants, understanding factors associated with their health service utilization will ultimately impact the health of the nation.

At present, China is actively developing the BPHS program and making significant progress. However, due to the imbalance of economic development in various regions of the country and large geographic gaps between urban and rural areas, BPHS equalization in China is relatively slow. For example, although health care record establishment is crucial for disease prevention and treatment, only 25.2% of female rural-to-urban migrants had established health care records in 2013 [5]. The establishment of health care records is still far lower than the 2013 National Health Commission goal of 65% [9]. Additionally, only 52.7% of female rural-to-urban migrants had received health education in 2014 [10], and a survey in Fujian province showed that the rate of breast and cervical cancer screening was only 38.2%, which was far below the rate for local residents (79.4%) [11].

Previous research has looked at several factors associated with health service utilization, but with inconsistent results. Compared to education below a primary school level, higher education was significantly associated with more health records establishment, health education utilization, adequate health literacy, maternal health service utilization, health examination and reproductive health consultation [12–15]. However, Liu et al. reported that education showed no significant association with reproductive health education and reproductive health examination for migrants [16]. This was corroborated by Wang et al. who reported that migrant education level had no association with utilization of health education [17].

Social support refers to the perception and status of a person being cared for and receiving help from others [7]. High levels of social support are hypothesized to encourage utilization of preventive services [10, 18]. Hou et al. reported that participating in social organizations and activities was associated with significantly more health record establishment and health education utilization by migrants compared to those who did not participate in these activities [10], which indicates that social connections may have a positive influence on BPHS utilization. Migrants who followed migrating family members were significantly more likely to establish health records [19] than those who traveled alone to the migration city, which also indicates the importance of social support. However, there are very few studies reporting the relationship between social support and BPHS utilization among rural-to-urban migrants and no study that uses a social support scale to explore the relationship between specific types of social support and BPHS utilization. Since female rural-to-urban migrants (even if married) are a unique group and are often separated from family and friends, having few links with a new community may weaken their social support network. Therefore, exploring how multi-dimensional social support affects BPHS utilization is necessary.

Finally, since migrants are often unfamiliar with their working and living environments, they can be marginalized and experience both internal and external discrimination [20–23]. As a result, experiences of discrimination may further impact BPHS utilization [18]. To our knowledge, there is no study reporting the relationship between discrimination and BPHS utilization. However, Hausmann et al., in the US, found that perceived discrimination was significantly associated with lower utilization of certain preventive measures including mammograms, pap tests, and colonoscopies [24]. Without understanding the impact these factors have on BPHS utilization among married female rural-to-urban migrants, the needs of this group will remain unknown, and thus leave the women vulnerable to adverse health outcomes.

Although, several studies have explored factors related to BPHS utilization in rural-to-urban migrants, most studies have included only demographic and migration factors, whereas social structural factors like social support and discrimination are rarely taken into account [10]. According to the Andersen Behavioral Model [25], social structure factors, that encompass personal, family, and community factors, affect health care services utilization. In our study, we include social support, discrimination, years of residence, length of migration, and living circumstances as social structure factors to examine its relationship with BPHS utilization. Also, there are inconsistencies in the relationship between socio-economic status (SES) and BPHS utilization. Our model of BPHS utilization fills this gap by using Anderson’s Behavioral model as a framework for considering important predisposing characteristics and enabling factors of health behavior. Therefore, the purpose of this study is to explore how demographic and social structure factors influence BPHS utilization among rural-to-urban, married migrant women. The specific aims are to: (1) describe frequency of utilization of BPHS services in rural-to-urban married migrant women, and (2) examine how social support, age, education, income, years of residence, length of migration, living circumstances, family location, and discrimination influence this utilization.

## Methods

### Study sites and recruitment

This study utilized a cross-sectional method to examine the BPHS utilization of married female rural-to-urban migrant workers in Changsha, Hunan province, the People’s Republic of China. Hunan Province, located in central China, has the second largest internal migrant population in China with an estimated 16.8 million in 2013, accounting for about 31% of the adult population [26]. Changsha is the capital and the most populous city of Hunan Province; in 2016, Changsha had 7,645,200 residents [26]. The city is an important commercial, manufacturing and transportation center [27]. In this study, 380 female migrant workers were originally recruited by trained, Mandarin-speaking research assistants from two medical device factories, two high-tech factories, a restaurant, a recycling factory, an automotive parts store, a textile factory and a hospital (custodial workers) in Changsha. We chose these locations because migrant women are concentrated in these labor-intensive industries. Of the 380 migrant women recruited, 343 completed a questionnaire and after data cleaning, 307 participant surveys were included in the study. Inclusion criteria included women: (1) 18–50 years of age, (2) married (basic contraceptive services are provided only for married couples), (3) with rural *hukou* and who had moved to an urban city for work, and (4) living in Changsha as a migrant worker for at least one month.

### Study procedure

Verbal informed consent was sought from all the participants after providing a description of the study. A researcher read the details of the verbal consent information sheet including the aim, risks and benefits of the study, what will happen if they take part in this study, and letting them know they can withdrawal from the study at any time without penalty. Participants were provided a survey and pen and a private area to complete the surveys. Research assistants were present to answer questions. The survey took about 30 min to complete. A small present was given to the participants who completed the survey. Approval for this study was obtained from the Ethics Committee of the Xiangya Nursing School, Central South University.

### Measurement

An author-designed survey was developed to collect data about the women’s demographic and social structure information (age [in years], family location [village or county; a county is an administrative region one level higher than a village], time in Changsha as a migrant [≤ 2 years, > 2 years], total time as migrant [< 4 years, ≥ 4 years], education [primary, middle/high school, diploma, or college], monthly income [≤ 3000 RMB or > 3000RMB] and living circumstances [separate from family or living with family]). This is provided as Additional file 1.

Social support was measured using the Social Support Rate Scale (SSRS) developed by Xiao [28], which has shown good reliability among Chinese populations [29]. The SSRS is a 10-item scale measuring three dimensions of social support: subjective social support, objective social support, and utilization of social support [28]. Questions on subjective social support include four questions: (A) How many friends do you have who can provide help and support to you? (1) None, (2) 1–2, (3) 3–5, (4) 6 and above; (B) How is your relationship with your neighbors? (1) only bowing acquaintance, (2) care a little when you are in trouble, (3) some neighbors care about you, or (4) most of the neighbors care about you; (C) How is your relationship with your colleagues? (1) only bowing acquaintance, (2) care a little when you are in trouble, (3) some colleagues care about you, or (4) most of the colleagues care about you; (D) How is the support you receive from your family members (spouses, parents, children, siblings, other family members)? (1) None, (2) seldom, (3) sort of support, or (4) full support [28]. Questions on objective social support include three questions: (A) What was your living condition in the past year? (1) away from family and lives alone, (2) accommodation is constantly changing, most of the time living with strangers, (3) lives with classmates, colleagues or friends, or (4) lives with families; (B) What have been your sources of economic and practical support in case of emergency? (1) no sources, (2) the following sources (multiple choice): (a) spouse, (b) other family members, (c) relatives, (d) colleagues, (e) employer/company, (f) official or semi-official bodies (e.g. political parties, or a labor union) (g) unofficial bodies (e.g. religious groups, social groups or other social organizations), or (h) others (please identify); (C) What have been your sources of care when you were in trouble? (same options above) [28]. Questions on the utilization of social support include three questions: (A) What is your way of confiding when you are in trouble? (1) never talk to anyone, (2) only talk to one or two intimate friends, (3) if friends ask, I will say, or (4) Take the initiative to talk about my problems in order to gain support and understanding; (B) How do you seek help when you are in trouble? (1) depend on my own and turn to no one, (2) seldom turn to others for help, (3) sometimes seeks help from others, or (4) turns to family, relatives and organizations; (C) To what extent are you involved in group (e.g. political organizations, religious organizations, labor unions and student unions) activities? (1) Never, (2) seldom, (3) often, or (4) actively involved. A higher score indicates greater social support (Cronbach’s α = 0.68). The possible total score of the SSRS ranges from 12 to 66 points [28].

Self-perceived discrimination was measured using a six-items scale developed by Chen [30]. The scores on the first three items (A harder time finding work than others; A harder time getting medical care; A harder time getting kids into local schools.) quantify perceived institutional discrimination (Cronbach’s α = 0.68) [30]. The scores on the last three items (You notice other people avoiding you; People are more impolite to you than others; People call you names or insult you.) measure perceived interpersonal discrimination (Cronbach’s α = 0.72) [30]. Both measures range from 1 to 5, with 1 indicating “never” and 5 indicating “very often.” [30].

The utilization of four basic women’s health services was measured with dichotomous “yes/no” questions. Health care record establishment was defined as whether or not a migrant woman had completed health records in a CHC. Utilization of health education was defined as whether or not the migrant woman had received health education at least once from a CHC. Utilization of free contraceptive services was defined as whether or not a migrant woman had received at least one free condom from a CHC, and utilization of breast and cervical cancer screening was defined as whether or not a migrant woman had received at least one breast or cervical cancer screening examination. (The BPHS questionnaire developed for this study is provided as Additional file 2.) Because we were interested in the potential effects of migration factors on BPHS utilization, we eliminated prenatal diagnosis evaluation and maternal health care services as outcome criteria because these two services were not applicable to all participants in our study. We did not ask participants if they had ever been pregnant or if they had been pregnant before or after migration to Changsha. If migrants had given birth in their rural hometown, they would not have used prenatal diagnosis evaluation and maternal health care services in the CHC.

### Data analysis

Frequency and descriptive statistics were obtained using percent for all dichotomous variables and means and standard error for continuous variables. Associations between family location, migration time in Changsha, migration time in total, years of education, monthly income and living circumstances and BPHS utilization (health care record establishment, health education, free contraception and cancer screening) were obtained using Chi-square tests. Associations between age, institutional discrimination, interpersonal discrimination, objective social support, subjective social support, and utilization of social support for the abovementioned BPHS utilization were conducted by univariate logistic regression with resultant odds ratios (ORs) and 95% confidence intervals (CIs).

Multivariate logistic regression analyses were performed to examine factors significant for each individual BPHS in Chi-square tests and univariate logistic regression for utilization of the four BPHS while controlling for demographic variables significantly associated with BPHS. We used enter methods to select variables correlated with BPHS utilization with resultant odds ratios (ORs) and 95% confidence intervals (CIs). The inclusion P value was 0.05, and the removal P value was 0.10. The data was analyzed using SPSS 18.0.

## Results

Table 1 displays descriptive statistics on measures of BPHS utilization, social support, discrimination, migration-related factors, and socio-demographic characteristics. The mean age was 35 years and participants were more likely to have completed middle or high school (68.6%) compared to primary school (4.9%) or college (10.8%). Approximately 63.5% earned less than 3,000 RMB in monthly income and 67.2% originally migrated from a rural village (compared to a county). Participants were more likely to be living separately from their families (75.6%), having worked at least two years as a migrant in Changsha (78.1%), and at least four years as a migrant worker in general (67.2%).

**Table 1:** (a) Percent distributions of sociodemographic characteristics and health service utilization and (b) means, standard deviations and ranges for quantitative indicators of discrimination and social support

Characteristic	Percent
*a) Percent distributions of sociodemographic characteristics and health service utilization N = 307*	
Family location	
Village	67.2
County (one level up from village)	32.8
Migration time in Changsha	
≤ 2 years	21.9
> 2 years	78.1
Migration time in total	
< 4 years	32.8
≥ 4 years	67.2
Years of education	
Primary	4.9
Middle/high	68.6
Diploma	15.7
College	10.8
Monthly income	
≤ 3000 RMB	63.5
> 3000 RMB	36.5
Living circumstance	
Separate from families	75.6
Living with families	24.4
Health record establishment (% Yes)	27.2
Health education (% Yes)	24.7
Basic contraceptive services (%Yes)	40.9
Breast and cervical cancer screening (%Yes)	26.1

Quantitative indicator	Mean (SD)
*(b) Means, standard deviations and ranges for quantitative indicators of discrimination and social support*	
Age in years	34.87 (6.31)
Institutional discrimination	5.30 (2.08)
Interpersonal discrimination	4.45 (1.64)
Objective social support	8.68 (3.00)
Subjective social support	24.91 (4.46)
Utilization of social support	7.22 (1.78)

*SD* standard deviations

For measures of BPHS utilization, health education among married migrant women was reported to be the lowest (24.7%), followed by breast and cervical cancer screening (26.1%), establishment of health care records (27.2%); the highest utilization was for basic contraceptive service (40.9%). Compared to the mean interpersonal discrimination score (4.45, range 3–15), female migrants perceived a higher level of institutional discrimination (5.30, range 3–15). The mean for objective social support, subjective social support and utilization of social support were 8.68 (range 1–22), 24.91 (range 8–32), 7.22 (range 3–12), respectively.

Table 2 show the relationship between BPHS utilization and family location, years of residence, migration time in total, migration time in Changsha, living circumstances, income, and education using a Chi square test. Migrant women who had lived in a county (one level higher than village), having less than four years migration experience, and living with their families were more likely to establish health care records. Migrant women who had lived in a county, and living with their families were more likely to receive health education. Migrant women who had lived in a county, with a primary, middle or high school education, having more than two years residence in migration city, and living with their families were more likely to receive basic contraceptive services. Migrant women with a primary, middle or high school education, living with their families and whose monthly income was less than 3000 RMB were more likely to receive cancer screening.

**Table 2:** Results of Chi-square test and descriptive statistics for utilization of basic public health services by socio-demographic factors among rural-to-urban migrant, married, women (*N* = 307)

	Health records establishment		Health education		Basic contraceptive services		Cancer screening	
	Utilized	Chi-square (*p*)	Utilized	Chi-square (*p*)	Utilized	Chi-square (*p*)	Utilized	Chi-square (*p*)
*Family location*		3.95 (0.047)		4.11(0.043)		8.45 (0.004)		3.73 (0.053)
Village	48 (23.5%)		43 (21.3%)		72 (35.5%)		47 (22.9%)	
County	34 (34.3%)		32 (32.0%)		52 (53.1%)		33 (33.3%)	
*Migration time in Changsha*		1.30 (0.255)		< 0.01(0.964)		4.71 (0.030)		< 0.01 (0.974)
≤ 2 years	14 (21.5%)		16 (24.6%)		19 (29.2%)		17 (25.8%)	
> 2 years	67 (28.6%)		58 (24.9%)		103 (44.2%)		61 (26.0%)	
*Migration time in total*		9.08 (0.003)		3.71 (0.054)		1.77 (0.184)		1.67 (0.196)
< 4 years	38 (38.4%)		31 (31.6%)		45 (46.4%)		30 (30.6%)	
≥ 4 years	44 (21.9%)		43 (21.4%)		77 (38.3%)		48 (23.6%)	
*Living circumstance*		9.34 (0.002)		15.61(< 0.001)		9.37 (0.002)		12.54 (< 0.001)
Separate with families	53 (22.8%)		44 (19.1%)		82 (36.0%)		49 (21.1%)	
Living with families	30 (41.1%)		31 (41.9%)		42 (56.0%)		31 (41.9%)	
*Monthly income*		0.09 (0.760)		0.21 (0.647)		3.66 (0.056)		6.34 (0.012)
≤ 3000 RMB	53 (27.7%)		49 (25.8%)		86 (45.5%)		60 (31.2%)	
> 3000RMB	29 (26.1%)		26 (23.4%)		38 (34.2%)		20 (18.0%)	
*Years of education*								
Primary/middle/high	60 (26.9%)	0.07 (0.797)	57 (25.7%)	0.38 (0.538)	102 (46.2%)	8.84 (0.003)	67 (29.9%)	5.91 (0.015)
Diploma/college	23 (28.4%)		18 (22.2%)		22 (27.2%)		13 (16.0%)	3.73 (0.053)

Table 3 shows the relationships between BPHS utilization and discrimination, social support, and age using univariate logistic regression. The study demonstrated that the more objective social support married migrant women have, the more likely they are to have received health education services (OR = 1.12; 95% CI = 1.02, 1.23; *p* = 0.014); however, there was no significant relationship between objective social support and health record establishment or basic contraception and cancer screening utilization. For subjective social support, there was a 9% increase in the odds for a one-unit increase in utilization of free contraceptive services (OR = 1.09; 95% CI = 1.03, 1.15; p = 0.004) and cancer screening (OR = 1.09; 95% CI = 1.02, 1.16; *p* = 0.009), but not significant for health record establishment and health education. Utilization of social support was significantly associated with establishment of health records (OR = 1.24; 95% CI = 1.06, 1.44; *p* = 0.006), basic contraceptive services (OR = 1.27; 95% CI = 1.10, 1.47; *p* = 0.001), and cancer screening service utilization (OR = 1.30; 95% CI = 1.11, 1.52; *p* = 0.001), although, not for having received health education. Discrimination and age were not significantly associated with BPHS utilization.

**Table 3:** Results of univariate logistic regression for basic public health services by discrimination, social support, and age among rural-to-urban married migrant women (*N* = 307) in Changsha, Hunan Province, China

	Health records establishment		Health education		Basic contraceptive tool		Cancer screening	
	OR	95% CI	OR	95% CI	OR	95% CI	OR	95% CI
*Discrimination*								
Institutional	0.96	0.83–1.11	1.00	0.87–1.16	0.90	0.79–1.02	0.90	0.78–1.05
Interpersonal	0.89	0.73–1.06	0.96	0.80–1.15	1.07	0.91–1.26	1.03	0.86–1.24
*Social support*								
Objective	1.08	0.99–1.18	1.12	1.02–1.23*	0.99	0.90–1.06	0.96	0.88–1.05
Subjective	1.04	0.98–1.11	1.05	0.98–1.12	1.09	1.03–1.15**	1.09	1.02–1.16**
Utilization	1.24	1.06–1.44**	1.14	0.98–1.34	1.27	1.10–1.47**	1.30	1.11–1.52**
Age	0.99	0.95–1.03	0.99	0.95–1.04	0.99	0.96–1.03	1.02	0.98–1.06

*OR*  odds ratio, *CI *confidence interval

*P < 0.05; **P < 0.01

Table 4 presents factors significantly associated with BPHS resulting from Chi square tests (living circumstances, migration time in total, migration time in Changsha, years of education,

**Table 4:** Results of multivariate logistic regression for basic public health services by living circumstances, social support, and education among rural-to-urban married migrant women (*N* = 307) in Changsha, Hunan Province, China

	Health records establishment		Health education		Basic contraceptive service		Cancer screening	
	OR	95% CI	OR	95% CI	OR	95% CI	OR	95% CI
Family location	1.33	0.75–2.35	1.48	0.84–2.60	1.96	1.12–3.44*		
Migration time in Changsha					2.24	1.18–4.27*		
Migration time in total	0.53	0.31–0.92*						
Living circumstances	2.11	1.17–3.80*	2.71	1.51–4.87**	1.59	0.88–2.90	2.38	1.30–4.36**
*Social support*								
Objective			1.15	1.04–1.26**				
Subjective					1.11	1.05–1.18**	1.1	1.02–1.17**
Utilization	1.24	1.06–1.44**			1.21	1.04–1.42*	1.29	1.10–1.51**
Years of education					0.36	0.20–0.67**	0.68	0.30–1.52
Monthly income							0.57	0.28–1.17

*OR* odds ratio, *CI* confidence interval

*P < 0.05; **P < 0.01

monthly income, and family location) and univariate analysis (social support) in multivariate logistic regression analyses.

Living circumstances are positively associated with three BPHS services, but not with basic contraceptive service utilization. Compared with living separately from their families, married migrant women who lived with their families were more likely to establish health record (OR = 2.11; 95% CI = 1.17, 3.80), to have received health education (OR = 2.71; 95% CI = 1.51, 4.87), and to have received cancer screening (OR = 2.38; 95% CI = 1.30, 4.36).

Utilization of all BPHS services was associated with social support. The more objective social support married migrant women had, the more likely they were to have received health education (OR = 1.15; 95% CI = 1.04, 1.26). However, utilization of health record establishment, cancer screening, and basic contraceptive services were not significantly associated with objective social support. The more subjective social support married migrant women had, the more likely they were to have received basic contraceptive services (OR = 1.11; 95% CI = 1.05, 1.18) and cancer screening (OR = 1.10; 95% CI = 1.02, 1.17). However, utilization of health record establishment and health education was not significantly associated with subjective social support. Finally, the more married migrant women utilized social support, the more likely they were to establish a health care record (OR = 1.24; 95% CI = 1.06, 1.44), to have basic contraceptive tools (OR = 1.21; 95% CI = 1.04, 1.42) and cancer screening (OR = 1.29; 95% CI = 1.10, 1.51). There was no significant association between social support and health education.

For education and monthly income, only education showed associations with received basic contraceptive services. Married female migrants with a diploma or college education were 64% less likely to have received basic contraceptive services (OR = 0.36; 95% CI = 0.20, 0.67) compared to those with lower levels of education.

Family location and Migration time in Changsha only associated with basic contraceptive services utilization among four BPHS. Compared to married female migrants who originated from a village, those from a county were 1.96 times more likely to have received basic contraceptive services (OR = 1.96; 95% CI = 1.12, 3.44). Married female migrants who had lived in Changsha for more than two years had received significantly more basic contraceptive services (OR = 2.24; 95% CI = 1.18, 4.26) than those who had lived there less time. Overall migration time only associated with health record establishment among four BPHS. Married female migrants with more than four years of migration in total were 47% less likely to have health record establishment (OR = 0.53; 95% CI = 0.31, 0.92).

## Discussion

This study examined BPHS utilization rates among rural-to-urban, married female migrants and the associations between social support, discrimination, and sociodemographic factors and BPHS utilization. Results showed that for all BPHS, rural-to-urban married female migrants had the lowest rates for health education utilization and the highest rates for basic contraceptive utilization. However, these rates are still far from sufficient. For the second study aim, results support a strong relationship between higher perceived social support and utilization of all four BPHS. Rural-to-urban married female migrants who live with their families were more likely to utilize BPHS health care records establishment, health education, and cancer screening.

Overall, rural-to-urban married migrant women have inadequate BPHS utilization according to our study. The establishment of health care records is still far lower than the 2014 National Health Commission goal of 70% [31]. Importantly, establishment of a health record is a basic service offered to all individuals and the required first step to receive other medical services, for example, chronic disease or cancer screening. If migrants seek help from CHC, they need to first establish a health care record, therefore, the low rate of health record establishment in our study indicates that married female rural-to-urban migrants have low medical service utilization.

After univariate regression, only seven factors demonstrated significance on the utilization of one or more BPHS. First, we found that married migrant women living with their families have significantly better BPHS utilization compared with those who live separately. The same pattern was also found for migrants in other countries. In a study of Latinos in the US, Mulvaney-Day [32] found that after controlling for demographic indicators, only family support had an association with self-rated physical health, while friend support and neighborhood social cohesion showed no significant relationship with self-rated physical health. In China, a study by Chen [33] found that migrating without a spouse had a negative effect on a migrant’s health, and in another study in China, migrant women’s separation from their children had a significant impact on their migration stress [34]. Therefore, it is essential for health care providers to focus on migrants who migrate alone, and relevant policy should encourage co-migration to maintain migrants’ health.

Second, previous studies have shown that social support has a positive effect on health for female migrants [35, 36]. To our knowledge, there are no studies using scales to measure social support to explore the relationship between social support and BPHS utilization in China, however, studies have included proxies for social support including social participation. Hou’s study in China [10] found that migrants who participated in social activities and organizations had higher rates of heath record establishment and health education than those who had not, which is consistent with our study’s finding that social support had a positive relationship with married female migrants’ health record establishment, health education, free contraception utilization, and cancer screening. Subjective social support and utilization of social support showed positive associations with three BPHS (but not health education), which indicates that married female migrants’ perceived social support and use of social support impacted their BPHS utilization more compared to objective social support. Even if female migrants have a great deal of social support, without a perceived sense of support or engaging in social support, the women’s BPHS utilization will remain inadequate. Therefore, health care providers should focus on organizing activities that can promote female migrants use of social support. Providers should also build trust with female migrants to enhance their sense of social support, especially for those who feel lonely.

Finally, our study demonstrated that higher levels of education were associated with lower utilization rates of basic contraceptive services by married female migrants. It is important to note that in this study, BPHS were provided only by CHC, which is likely the result of the National Health Commission’s goal of encouraging development of BPHS in these settings. However, previous studies examining BPHS utilization often focus on specific services like reproductive health services, which may also be provided in specialty hospitals and family planning service centers [16]. It is important to note that contraceptives may also be acquired in pharmacies without having to consult a health care provider. Therefore, the association between level of education and utilization of contraceptive services may be explained by the fact that migrants with higher levels of education may self-select out of the community health system in favor of more comprehensive care medical institutions like specialty hospitals. Finally, after multivariate regression, there was no significant relationship between education and cancer screening.

In our study, perceived discrimination did not account for low BPHS utilization among rural-to-urban, married female migrants. There are no studies that have examined the relationship between perceived discrimination and BPHS in China, although studies conducted in the US have corroborated that perceived discrimination is negatively related to visits for management of chronic disease, but not for receiving cancer screening [37–39]. Our findings are consistent with this, since we found that screening for breast and cervical cancer were unrelated to whether married migrant women perceived institutional or interpersonal discrimination.

This study demonstrated that effective interventions to promote health-seeking behaviors in rural-to-urban, married female migrants in China. We found that enhancing utilization of social support is an essential factor in utilization of BPHS in China. This could be accomplished by encouraging female migrants to attend social activities with friends and family members in order to promote perceived social support. It would also be helpful to encourage female migrants, who are alone and new to a city, to attend activities and thereby build their social network to enhance perceived social support. Health care providers can also enhance their communication with female migrants by offering outreach services, for example, to further increase the women’s perceived social support and, as a result, increase utilization of BPHS.

Stakeholders should focus on female migrants who migrate from villages, travel far to their migration city, and are recent migrants to a city. It may be necessary for neighborhood committees and CHC from various jurisdiction to collaborate and share access and contact information with female migrants. This can be accomplished by having community neighborhood committees obtain the women’s migration-related information through door-to-door visits and share this information with the CHC within the jurisdiction. If female migrants are going to migrant to other jurisdiction or cities, CHC could then forward the women’s health information to the corresponding jurisdiction. Besides, health care providers can also increase the frequency of phone contact with female migrants to track their mobility and inform them about BPHS in CHC.

In summary, as evidenced, health care providers are essential to both identifying migrants at risk for low utilization of BPHS and implementing programs that will increase use of BPHS. It will be important to plan future studies that examine the efficacy of such programs to improve our understanding regarding use of these vital resources.

## Strengths and limitations

This study has several important strengths. First, to our knowledge, this is the first study to explore the relationship between multi-dimensional social support and BPHS utilization among migrants. Rural-to-urban married female migrants perceived social support and family support as essential to influencing their BPHS utilization. We incorporated the concept of social support in an empirical framework and assessed the relationship between social support and health care utilization. Second, this is the first study to examine the relationship between perceived discrimination and BPHS utilization among married female migrants.

This study also has limitations. First, the sample was not selected from a city-wide random sample, which limits the generalizability and application of its findings. Future studies should include several geographic locations and samples sizes, as well as multiple work sites. Second, this cross-sectional study design limited our ability to find true causes of underutilization. Future studies should employ longitudinal designs to explore how the experience of social support, discrimination, and migration-related factors change over time and affect BPHS utilization. Third, recall bias is inherent in this study since the women had to recall previous BPHS utilization.

## Conclusion

In our study, we found an overall low utilization rate for all four BPHS among rural-to-urban, married female migrants. Most importantly, were the low health record establishment rate (necessary for migrants to receive health care services in CHC) and low health education utilization rate (vital channel for migrants to gain access to the BPHS information and policies). Therefore, focused efforts should be made to improve this process of establishing care and receiving health education.

Our study found that family support and perceived social support play an important part in rural-to-urban, female married migrants’ use of BPHS. Education is not associated with major BPHS utilization, which means health care providers should focus on all migrants regardless their education level. Institutional discrimination has a negative association with BPHS use although, further study to explore the significance of their relationships among rural-to-urban, married female migrants is needed. Therefore, several suggestions have been made to target rural-to-urban, married female migrants according to four specific BPHS in order to improve utilization of BPHS in CHC.

## Supplementary Information

**Additional file 1**. The questionnaire of women’s demographic and social structure information.

**Additional file 2**. The questionnaire of BPHS utilization.

## Data Availability

The datasets analysed during the current study are available from the corresponding author on reasonable request.

## Abbreviations

BPHS: Basic Public Health Services
CHC: Community Health Center
SSRS: Social Support Rate Scale
ORs: Odds ratios
CIs: 95% Confidence intervals
