# Supplementary material for: Frequency of basic public health services utilization by married female migrants in China: associations of social support, discrimination and sociodemographic factors
Source: BMC Womens Health. 2021 Sep 28;21:344. doi: 10.1186/s12905-021-01482-3 (PMC8480003; doi:10.1186/s12905-021-01482-3)
Supplement: Supplementary file 1 — Additional file 1. The questionnaire of women’s demographic and social structure information. [file 12905_2021_1482_MOESM1_ESM.docx]

1. How old are you?

_____

2. Where is your family location?

Village

County

3. How long have you lived in Changsha as a migrant?

≤ 2 years

＞ 2 years

4. How long have you been a migrant? (Total time as a migrant)

＜4 years

≥4 years

5. What is the highest level of school you attended?

Primary

Middle or high school

Diploma or College

6. What is your average monthly income?

≤ 3000 RMB

＞ 3000RMB

7. What is your current living circumstance?

Separate from family

Living with family
