# Supplementary material for: Frequency of basic public health services utilization by married female migrants in China: associations of social support, discrimination and sociodemographic factors
Source: BMC Womens Health. 2021 Sep 28;21:344. doi: 10.1186/s12905-021-01482-3 (PMC8480003; doi:10.1186/s12905-021-01482-3)
Supplement: Supplementary file 2 — Additional file 2. The questionnaire of BPHS utilization. [file 12905_2021_1482_MOESM2_ESM.docx]

1. Have you ever completed health records in a community healthcare center?

Yes

No

2. Have you ever received health education at least once from a community healthcare center?

Yes

No

3. Have you ever received at least one free condom from a community healthcare center?

Yes

No

4. Have you ever received at least one breast or cervical cancer screening examination from a community healthcare center?

Yes

No
